# Common genetic variation in the Angelman syndrome imprinting centre affects the imprinting of chromosome 15

**DOI:** 10.1038/s41431-020-0595-y

**Published:** 2020-03-09

**Authors:** Jasmin Beygo, Christian Grosser, Sabine Kaya, Claudia Mertel, Karin Buiting, Bernhard Horsthemke

**Affiliations:** 1 Institut für Humangenetik, Universitätsklinikum Essen, Universität Duisburg-Essen, Essen, Germany; 2 Present Address: Praxis für Humangenetik Tübingen, Tübingen, Germany

**Keywords:** Epigenetics, Epigenomics

## Abstract

Angelman syndrome (AS) is a rare neurogenetic imprinting disorder caused by the loss of function of *UBE3A*. In ~3–5% of AS patients, the disease is due to an imprinting defect (ID). These patients lack DNA methylation of the maternal *SNRPN* promotor so that a large *SNRPN* sense/*UBE3A* antisense transcript (*SNHG14*) is expressed, which silences *UBE3A*. In very rare cases, the ID is caused by a deletion of the AS imprinting centre (AS-IC). To search for sequence alterations, we sequenced this region in 168 patients without an AS-IC deletion, but did not detect any sequence alteration. However, the AS-IC harbours six common variants (five single nucleotide variants and one TATG insertion/deletion variant), which constitute five common haplotypes. To determine if any of these haplotypes is associated with an increased risk for an ID, we investigated 119 informative AS-ID trios with the transmission disequilibrium test, which is a family-based association test that measures the over-transmission of an allele or haplotype from heterozygous parents to affected offspring. By this we observed maternal over-transmission of haplotype H-AS3 (*p* = 0.0073). Interestingly, H-AS3 is the only haplotype that includes the TATG deletion allele. We conclude that this haplotype and possibly the TATG deletion, which removes a SOX2 binding site, increases the risk for a maternal ID and AS. Our data strengthen the notion that the AS-IC is important for establishing and/or maintaining DNA methylation at the *SNRPN* promotor and show that common genetic variation can affect genomic imprinting.

## Introduction

**Fig. 1:**
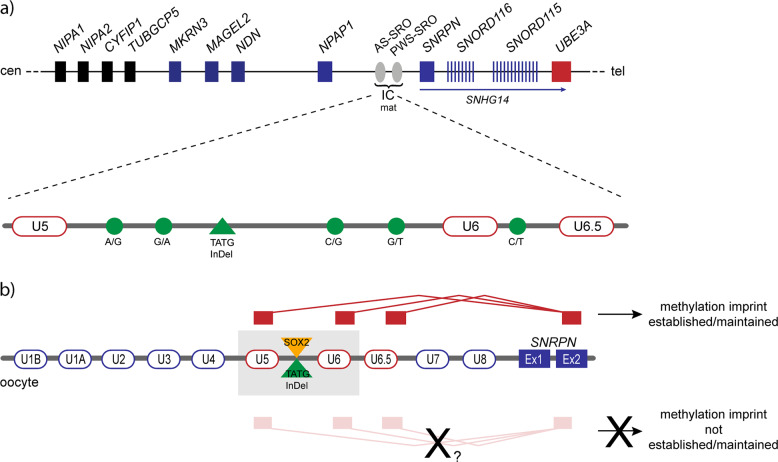
Overview of the imprinted gene cluster on chromosome 15q11q13 and the AS-IC and possible effect of the haplotype HAS-3. **a** The figure gives an overview of the genes within the imprinted cluster on chromosome 15q11q13. Genes depicted in blue are paternally expressed, the *UBE3A* gene is depicted in red for maternal only expression (situation in neurons). The biallelically expressed genes are shown in black. The bipartite structure of the IC is shown with the light grey ovals indicating the AS- and the PWS-IC. The AS-IC region is shown expanded below including the oocyte-specific transcription start sites at the upstream exons U5, U6 and U6.5 (rounded rectangles) and the six common variants (green circles for single nucleotide substitutions, a green triangle for the 4 bp InDel). **b** The figure shows part of the *SNRPN* gene with some of its upstream exons (rounded rectangles). Upstream exons U5 and U6 are located within the AS-IC (grey region) and U6.5 is located directly 3′. All three upstream exons have been shown to be oocyte-specific transcription start sites indicated by their red colour [5]. The transcripts originating at these exons all splice onto exon 2 of the *SNRPN* gene (blue rectangle), indicated above. The 4 bp InDel variant (green triangle), that is only present in haplotype H-AS3, affects a SOX2 binding site (orange triangle; [18]). This variant might affect SOX2 binding and - possibly in conjunction with other factors - could have an effect that hinders establishment of the methylation imprint at the AS-IC. Not drawn to scale. cen centromeric, tel telomeric, IC imprinting centre, mat maternally methylated.

Angelman syndrome (AS, #105830) is a rare neurogenetic imprinting disorder characterised by severe intellectual disability with absence of speech, microcephaly, ataxia, seizures and a happy demeanour. It is caused by genetic and epigenetic alterations on chromosome 15q11q13 leading to loss of expression of the imprinted gene *UBE3A*, which in neurons is expressed from the maternal allele only [1] (Fig. 1a). In about 3–5% of AS patients, the disease is due to an imprinting defect (ID). These patients have biparentally inherited chromosomes 15, but the maternal allele carries a paternal epigenotype and is unmethylated. Lack of DNA methylation at the *SNRPN* promotor (*SNURF*:TSS-DMR [2]) leads to the expression of a large *SNRPN* sense/*UBE3A* antisense transcript (*SNHG14*) on the maternal allele, which silences *UBE3A* [1]. In very rare cases the ID is caused by a deletion of the AS imprinting centre (IC; previously termed AS-SRO for smallest region of deletion overlap [3, 4]) and associated with a recurrence risk of 50% if the mother is a non-mosaic carrier. In contrast, in families with non-IC-deletion AS-ID patients the recurrence risk does not seem to be elevated. The AS-IC has a size of about 880 bp and is located ~35 kb upstream of the *SNRPN* promotor. The AS-IC is responsible for the establishment of the maternal imprint in the female germ line [3]. Recently, oocyte-specific transcription start sites at *SNRPN* upstream exons located within the AS-IC were identified, demonstrating that the AS-IC serves as an oocyte-specific promotor [5]. The transcripts originating here or the process of transcription through the locus is believed to be necessary for the establishment of the maternal methylation imprint [5–7]. Similar observations have been made at four other imprinted loci: *GNAS/Gnas* [8], *KCNQ1*/*Kcnq1* [9–12], *Peg3* [13] and *Zrsr1* [14]. Therefore, sequence alterations in the promoter or upstream exons located within the AS-IC might affect transcription in this region and thereby the establishment of the methylation imprint. To investigate this possibility, we performed Sanger sequencing of this region in a total of 168 AS-ID patients. Furthermore, following up on our previous study [15], we investigated the transmission of the five common haplotypes in a new, larger cohort of AS-ID trios and in a combined cohort, consisting of 48 AS-ID trios from the Zogel study and 72 new AS-ID trios. So far, common genetic variation had only been implicated in another imprinting disorder, the Beckwith–Wiedemann syndrome [16].

## Material and methods

### Material

All patients have a molecular diagnosis of AS due to an ID. Older cases had been identified using methylation-specific (MS)-PCR [15], newer cases by MS-MLPA (methylation-specific multiplex ligation-dependent probe amplification, kit ME028, different versions, MRC Holland, Amsterdam, Netherlands). IC-deletions were excluded either by MS-MLPA or in older cases by quantitative real-time PCR. Uniparental disomy of chromosomes 15 was excluded in all cases by microsatellite analyses.

To the best of our knowledge, none of the AS-ID patients in this study had affected siblings.

In our former study, 48 AS-ID trios were described [15]. Informed consent was obtained for the so far unpublished participants. The study was approved by the ethics committee of the University Duisburg-Essen (AZ 08–3858).

The data has been submitted to the LOVD database (https://urldefense.proofpoint.com/v2/url?u=https-3A__databases.lovd.nl_shared_references_DOI-3A10.1038_s41431-2D020-2D0595-2Dy&d=DwIDaQ&c=vh6FgFnduejNhPPD0fl_yRaSfZy8CWbWnIf4XJhSqx8&r=FZZECvVW6LToxnfMxEanqbIvvLKGJ1qJ06OvmG6rJQw&m=wa5MA9FPSo0fZqkeit2NK63qqY5WxBmglJ02Z6IuDPw&s=TkrBfjHMyvmyTHm6y9e-WMEQsb5fIIdQScoJEFEZFxY&e=).

DNA was extracted from peripheral blood using the Flexigene Kit (Qiagen, Hilden, Germany) according to the manufacturer’s protocol or was obtained as extracted DNA.

The AS-IC region was amplified and sequenced using standard methods. PCR products were sequenced on an ABI3130XL capillary sequencer (Applied Biosystems). Data analysis was performed using the Sequencing Analysis (Applied Biosystems) and the Geneious software (Biomatters, Auckland, New Zealand). For details see Supplementary.

### Statistical analyses

Statistical analyses were performed as described before, i.e. the biallelic TDT [15] (see Supplementary). A goodness-of-fit-test for Hardy–Weinberg equilibrium showed no deviation (chi square ≤ 3.841).

## Results and discussion

First, we investigated whether ID in AS-ID patients without IC-deletions could be due to sequence alterations within the AS-IC and especially within the newly described oocyte-specific transcription start sites at the *SNRPN* upstream exons U5, U6 and U6.5 [5]. For this, we performed Sanger sequencing of this region in 168 AS-ID patients but did not detect any sequence alterations beside the known common variants. This suggests that nucleotide substitutions in this region are either extremely rare, not causative for AS-ID or are not tolerated.

The GnomAD database (https://gnomad.broadinstitute.org/; accessed August 2019) shows only variants with very low frequencies (MAF between 0.017 and 0.000031; average 0.0009046) within the AS-IC region with the exception of six common variants (five single nucleotide variants and one TATG insertion/deletion (InDel) variant), which constitute five common haplotypes [15, 17]. None of these common variants is located within a *SNRPN* upstream exon. We analysed if any of these haplotypes might be associated with an increased risk for an ID using the transmission disequilibrium test (TDT). The TDT is a family-based association test that measures the over-transmission of an allele or haplotype from heterozygous parents to affected offspring. Of note, test results are not affected by population structure. This is important, because the patients were recruited from many different countries. In 48 parent-child trios tested previously, we had detected a trend for maternal over-transmission of the haplotype H-AS3 (*p* = 0.058; Table 1a [15]). We have now analysed an additional cohort of 72 AS-ID trios and found the same trend for maternal over-transmission of the haplotype H-AS3 (*p* = 0.058; Table 1b) supporting the trend seen previously in the smaller cohort. By combining the data from both studies, we could extend the cohort to 119 AS-ID trios. Again, we observed maternal over-transmission of haplotype H-AS3 (*p* = 0.0073; Table 1c; for haplotype data see Supplementary Table 1). No other haplotype showed over-transmission from either parent.

**Table 1:** Results of the haplotype analyses.

Haplotypes	*p* values of the maternal transmission	Frequencies of the transmitted haplotype	Haplotypes	*p* values of the paternal transmission	Frequencies of the transmitted haplotype
(a) Revisited cohort [15] - 47 trios					
H-AS1	0.275^a^	0.381^a^	H-AS1	0.513^a^	0.429^a^
H-AS2	0.467^a^	0.412^a^	H-AS2	0.527	0.400
H-AS3	**0.058**	0.800	H-AS3	0.739	0.444
H-AS4	0.706	0.429	H-AS4	0.317	0.667
H-AS5	0.180	0.800	H-AS5	0.317	0.667
H-AS6	Not observed	Not observed	H-AS6	Not observed^a^	Not observed^a^
(b) New cohort this study - 72 trios					
H-AS1	0.398	0.429	H-AS1	0.223	0.394
H-AS2	0.695	0.462	H-AS2	0.371	0.600
H-AS3	**0.058**	0.800	H-AS3	0.808	0.529
H-AS4	0.248	0.667	H-AS4	0.527	0.400
H-AS5	0.317	0.333	H-AS5	0.157	0.750
H-AS6	Not observed	Not observed	H-AS6	Not observed	Not observed
(c) Combined cohorts - 119 trios					
H-AS1	0.181	0.411	H-AS1	0.174	0.407
H-AS2	0.446	0.442	H-AS2	0.715	0.533
H-AS3	**0.007**	0.800	H-AS3	1.000	0.500
H-AS4	0.491	0.579	H-AS4	0.819	0.526
H-AS5	1.000	0.500	H-AS5	0.090	0.706
H-AS6	Not observed	Not observed	H-AS6	Not observed	Not observed

After revisiting the data from the previous study and resequencing of single samples one trio was removed as the patient and both parents were heterozygous for the same haplotypes and thus the transmitted and non-transmitted haplotypes could not be assigned to the respective parent. In another trio a sequencing error led to the incorrect observation of H-AS6 in the father and the transmitted haplotype from the mother was assigned incorrectly.

*P*-values that show maternal over transmission or a trend towards maternal over transmission are highlighted in bold.

^a^Data have changed.

Interestingly, haplotype H-AS3 is the only haplotype that includes the TATG deletion allele. It has been shown that this deletion affects a SOX2 binding site [18]. SOX2 is a widely expressed transcription factor, which plays a crucial role in development [19, 20]. It is possible that the 4 bp deletion affects SOX2 binding and reduces transcription initiation at the AS-IC in the oocyte or early embryo. This may prevent imprint establishment or maintenance in some cells. (Fig. 1b). Since AS-IDs are extremely rare and we failed to see an effect of the TATG deletion in reporter gene assays in HEK293 cells (data not shown), additional *cis*- and *trans*-acting factors appear to be necessary for maternal imprinting in 15q11q13.

We conclude that DNA sequence alterations within the AS-IC do not seem to be a cause of AS-ID. This may explain why we have not seen AS-ID siblings in families without a maternal AS-IC deletion. However, the haplotype H-AS3 and possibly the TATG deletion, which removes a SOX2 binding site, seems to increase the risk for a maternal ID and AS.

In view of the fact that the population frequency of the H-AS3 haplotype is ~0.1 (see Supplementary) and the birth prevalence of a child with AS-ID is <1:1,000,000, the risk associated with this haplotype is too weak to warrant prenatal diagnosis.

It is also possible that the H-AS3 haplotype affects oocyte fitness. Due to sparse data we cannot investigate this at present. In the GnomAD database 66 women homozygous for the TATG deletion are listed but without further information. However, in our cohort, three mothers are homozygous for H-AS3 and all three have at least one unaffected child so that an effect, if present, cannot be severe.

In summary, this finding demonstrates that common genetic variation can affect genomic imprints and strengthens the notion that the AS-IC is important for establishing and/or maintaining DNA methylation at the *SNRPN* promotor.

## Supplementary information

Supplement

Supplementary Table 1
