# Supplementary material for: Common genetic variation in the Angelman syndrome imprinting centre affects the imprinting of chromosome 15
Source: Eur J Hum Genet. 2020 Mar 9;28(6):835–9. doi: 10.1038/s41431-020-0595-y (PMC7253442; doi:10.1038/s41431-020-0595-y)
Supplement: Supplementary file 1 — Supplement [file 41431_2020_595_MOESM1_ESM.pdf]

Supplement

Methods

The AS-IC region was amplified and sequenced as described before (Zogel et al. 2006). The newly identified oocyte-specific promotor region (Lewis et al. 2014) not completely included in the region investigated by Zogel et al. was amplified using standard protocols with primers AS-SRO_F_kurz 5’-ACAGTCCATGATTTGTCTGGTG-3’ and AS-SRO_R_k_u_l 5’-AGGCCAAGGGCTGATCATA-3’. In newer cases the whole region was amplified using primers IC16 5’-GCTCAAGCCGTGTTCATTTT-3’ (Zogel et al. 2006) and AS-SRO_R_k_u_l with additional primers for sequencing (usage depending on the presence of the 4bp InDel SNP rs36061418: AS-SRO_Seq_F 5’-GGTCAGCTGCTGTTAAAGGTT-3’, AS-SRO_Seq_R 5’-CAGCAGCTGACCTAAAACACA-3’, AS-SRO_Seq_F2 5’-GCTGGGGAGTAAGGGAGAAC-3’ and AS-SRO_Seq_R2 5’-AGCCTGTCAGAAGAGCCTTG-3’).

Sequencing of the PCR products was conducted on the ABI3130XL capillary sequencer (Applied Biosystems) with subsequent analysis using the Sequencing Analysis (Applied Biosystems) and the Geneious software (Biomatters, Auckland, New Zealand).

Transmission disequilibrium test (TDT)

The AS-IC haplotypes were determined as described previously (Zogel et al. 2006). Since the disease locus is subject to genomic imprinting, we determined the transmitted and non-transmitted haplotypes separately for each parent. Parents homozygous for a haplotype, or trios where the transmitting parent could not be determined (e.g. because all three family members shared the same two haplotypes), were excluded. TDT was calculated as described

by Spielman et al. 1996: $(b-c)^2/(b+c)$, where b is the number of times a given haplotype was not transmitted while c is the number of times the haplotype was transmitted. This was calculated separately for each haplotype and each parent. Significance of overtransmission was determined by chi square.

Investigated common variants

rs No.	Alleles	MAF	Genomic position (GRCh37.p13)
rs2075814	A/G	0.09922	NC_000015.9:g.25165776A>G
rs2736711	G/A	0.1649	NC_000015.9:g.25165844G>A
rs36061418	TATG/Del*	0.09835	NC_000015.9:g.25165936_25165939del
rs35207533	C/G	0.09915	NC_000015.9:g.25166089C>G
rs17114852	G/T	0.04958	NC_000015.9:g.25166183G>T
rs17785249	C/T	0.1684	NC_000015.9:g.25166455C>T

Haplotypes

H-AS1	A	G	TATG	C	G	C
H-AS2	A	A	TATG	C	G	T
H-AS3	G	G	Del	C	G	C
H-AS4	A	G	TATG	C	T	C
H-AS5	A	G	TATG	G	G	C

Haplotypes as given by Zogel et al. 2006. The order of the benign variants is the same as described above.

MAF – minor allele frequency

MAF data taken from GnomAD (the Genome Aggregation Database, https://gnomad.broadinstitute.org/ August 2019). The MAF data given in the table above correspond to all investigated samples given in GnomAD for the respective variants without differentiation of the investigated populations.

Variant identifiers (rs No.) and the genomic position according to GRCh37.p13 are taken from NCBI dbSNP version 153 (https://www.ncbi.nlm.nih.gov/snp/ August 2019).

* This variant was called TATG/Del up to dbSNP151. Afterwards it was changed to ATGT. For reasons of continuity, we stayed with TATG/Del.
